# Visual Fixation Patterns During Viewing of Half-Face Stimuli in Adults: An Eye-Tracking Study

**DOI:** 10.3389/fpsyg.2018.02478

**Published:** 2018-12-11

**Authors:** Ágoston Galambos, Borbála Turcsán, Katalin Oláh, Fruzsina Elekes, Anna Gergely, Ildikó Király, József Topál

**Affiliations:** ^1^Institute of Cognitive Neuroscience and Psychology, Research Centre for Natural Sciences, Hungarian Academy of Sciences, Budapest, Hungary; ^2^Department of Cognitive Psychology, Eötvös Loránd University, Budapest, Hungary; ^3^Department of Cognitive Science, Central European University, Budapest, Hungary

**Keywords:** face processing, inversion, complementary fixations, face template, fixation patterns

## Abstract

Human faces play a special role in social cognition, since as a core signal of interpersonal communication, they convey various kinds of information (e.g., about sex, age, race, emotions, intentions). Study 1 aimed to explore how this specialization manifests itself in eye movements when looking at neutral, static, female faces. We monitored the gaze pattern of 23 adult participants using eye-tracking method. To test if template-driven processes are involved in face perception, and to see how inversion affects fixations on special facial stimuli, we presented vertically cut half-faces in upright and inverted positions (so half of each stimulus represented a half-face, whereas the other half was left blank). Our results corroborate prior findings consistently demonstrating the dominance of the triangular area marked by the eyes and the mouth, measured by the number and duration of fixations. In addition, we found evidence for so-called complementary fixations, targeted at the non-informative parts (i.e., the half that does not contain any facial information) of the pictures, suggesting that other mechanisms beyond purely stimulus-driven ones might drive looking behavior when scanning faces. Study 2 was intended to test if these systematic eye movements are face-specific or occur in case of other visual objects as well.

## Introduction

Human faces are highly salient social stimuli that convey a wide range of socially relevant information (about sex, race, age, gaze direction, emotional state). According to the face-specificity hypothesis (see e.g., Kanwisher and Yovel, 2006), humans have a specific sensitivity to the perception of faces (“face-expert processor”: Barton et al., 2006); even newborns are predisposed to attend to and process face-like patterns (Valenza et al., 1996).

Faces have a typical configuration, yet they are highly variable. Although most objects that have a prevalent orientation are processed less efficiently when presented in an atypical fashion, this is particularly true for faces (a phenomenon called inversion effect, e.g., Haxby et al., 1999). Compared to normal, upright faces, inverted faces are extremely difficult to identify. Face-specificity theories (e.g., Kanwisher, 2000; McKone et al., 2007) agree that faces are processed in a different manner than other objects. Numerous studies (e.g., Goffaux and Rossion, 2006; Rossion, 2013) have shown that face perception is holistic rather than analytical^1^, resulting in a specific way of processing that goes beyond mere part-based perception. Faces are characterized by a certain structure that is composed of different parts, hierarchically organized (i.e., the parts are not equally effective in conveying information): one possible interpretation of the term “holistic” is that face processing involves the integration of features into a configuration (about the difficulties of defining “holistic,” see Richler et al., 2012). Following this definition, we may assume that there is a specific *Gestalt* (like the one described by Carbon and Leder, 2005: “an unmanipulated normal face”), a “prestored face model” (Sagiv and Bentin, 2001), a representational template for recognizing typical face-like configurations, and this template may modulate looking behavior when observing faces (i.e., complement feature-based processes). Based on this idea, scanning faces involves Gestalt-matching processes as well, which facilitate face recognition, for instance.

The putative existence of this Gestalt implies that the upright configuration may direct our fixations even in the absence of certain components of a given face. In other words, we may hypothesize that, when presented with an incomplete facial stimulus, the visually accessible, informative elements would not be the only ones we scan. We would fixate (at least partly) the non-informative^2^ but template-compatible parts as well. That is, on a (vertically cut) half-face, we would expect fixations at the empty half. We could also presume that these fixations would not be random but would rather correspond to the most salient parts of a face (where the eyes and the missing part of the nose and mouth should be). This is exactly what Dyer and Vassallo (2011) found. They presented participants with faces whose left side was covered (contained no visual information) whereas the right side showed a half-face after vertical cut. The results were the following: 64% of the participants made saccadic eye movements to the covered half, 45.7% of which were at the supposed position of the eye. These findings are truly compelling in light of the control trials: when participants were shown various objects, also cut in half, fixations at the covered half of the picture were limited. Throughout eight objects, only ten fixations were made at the empty half, compared to the informational one where even the less-fixated object evoked 159 fixations. The authors’ interpretation is that scanning faces (and not other objects) is at least partly template-driven. These processes might follow the typical, upright configuration of a face-template, directing most fixations at the eye region. Whereas a very promising study, we believed there was room for improvement. First, we wanted to measure the actual depth of visual processing, so we chose duration and number of fixations as dependent variables (instead of saccades). Second, in the above study, half-faces were presented only on the right side – we were curious about both sides (see below). Third, we considered the face-inversion effect to be of importance, therefore we included this kind of stimuli in our study. Hence, one objective of our study, using half-face stimuli (in upright/inverted orientation, presented on the left/right side), was to find out if there are, in fact, complementary fixations on an “empty,” non-informative part of a neutral, static face.

Although it is generally agreed that people can quickly form holistic representations of faces (e.g., Richler et al., 2009; Taubert et al., 2011), it is a valid question whether there is a hierarchy in the relevance of different parts of a face. Since the seminal work of Yarbus (1967), a growing body of research has shown that the eye and the mouth regions are the most informative parts of faces (e.g., Schyns et al., 2002; Eisenbarth and Alpers, 2011). The triangle marked by the eyes and the mouth constitute the region at which we look the most, and this corresponds well with our intuition, about the role these parts play in conveying social-communicative signals. Concerning the order of importance of the parts inside the triangle, results show a marked cultural effect. For example, Blais et al. (2008) compared the fixation patterns of 14 white and 14 East Asian participants using eye-tracking method. According to their findings, the former group fixated most at the eyes, then the mouth, whereas the latter group primarily scanned the central area of the face, around the nose region. Or et al. (2015), however, found only a minor cultural effect: analyzing the location of the first fixations, the authors concluded that both East Asian and Western participants tended to make the first eye movement somewhere between the eyes and the nose, with only a slight difference across cultures. Several other studies (e.g., Langton et al., 2000; Itier et al., 2006, 2007; Peterson and Eckstein, 2012) confirmed the special role of the eye region when looking at faces. Analysis of the fixation trajectory of participants using eye-tracking methodology has also corroborated the dominance of eyes (Henderson et al., 2005). Regarding the generalizability of the dominance of the eye-mouth triangle, Windhager et al. (2010) found that this pattern is present even when looking at inanimate objects. Their results showed that participants formed analogies between faces and cars, i.e., they scanned the corresponding parts of a car front when the task was to compare the eyes, nose, ears and mouth of a face and a car. When, for instance, they had to look at the “nose,” they fixated the grille of the car more than the air intake (mouth) or the side mirrors (ears). In addition, the “eyes” were of primary importance: even when they had to look for a nose in a car, for example, gaze was directed at the grille and the headlights (and this was enhanced when the task was to compare the eyes). These findings suggest that humans are prone to generalize the position of eyes (to other visual objects) and attribute a particular role to it – which results in increased attention to the place where the eyes “should be.”

In terms of the physical properties of faces (or face-like structures), there is empirical evidence that contrast polarity is of importance (e.g., a stable characteristic of eyes is a specific contrast-related arrangement: a darker element in a lighter area). Farroni et al. (2005) investigated newborns’ face processing ability with schematic and naturalistic face-like stimuli. Their major finding was that contrast polarity is important in determining whether a configuration is perceived as a face and hence if it elicits newborns’ preferential looking. Farroni et al. (2005) argue that what infants encounter when looking at faces typically consists of darker areas (eyes and mouth) on a lighter background – which is the result of normally occurring top-lit conditions. The findings can thus be interpreted that there might be an evolutionary preference toward a specific configuration that resembles an upright, top-lit face. These results suggest that certain physical aspects of faces have a tendency to elicit preference even from newborns when looking at face-like stimuli: (face-like) contrast polarity did play a role, whereas overall luminance or differences in general within-subject luminance did not. This is empirical evidence that stimulus-driven processes are indeed involved in face perception.

There is also ample evidence that face processing is neuroanatomically asymmetrical: the right hemisphere seems to show enhanced activity during perception of facial stimuli (e.g., Guo et al., 2009; Frässle et al., 2016). It is unclear, however, whether this merely reflects the right-hemisphere dominance for the neural processing of facial stimuli (Kanwisher et al., 1997), or the asymmetric nature of facial signals (i.e., asymmetry in facial expressions – Sackeim et al., 1978; Moscovitch and Olds, 1982) also play a role. Coutrot et al. (2016) found evidence for the left-gaze bias: interestingly, the effect was most pronounced when women participants were looking at female faces. Siman-Tov et al. (2007) showed that the left-gaze bias may not even be face-specific but rather there is an attentional bias toward left visual field stimuli in general. Presenting half-face stimuli on the left/right side would give insight into the subtleties of asymmetrical fixation patterns and would enable us to investigate the distribution of putative complementary fixations as a function of the side of presentation. Based on the above studies, one may expect to find differences between fixations targeted at the left and right half-faces. Namely, preference for the left half of the stimuli (from the same category): that is, more and longer fixations at the left compared to the right informative half, as well as the left compared to the right covered (empty) parts.

Therefore, in Study 1, we aimed to gather eye-tracking data regarding (i) the asymmetrical processing of neutral, static, female facial stimuli (leftward bias), (ii) the differences in fixations at the socially relevant (eye, nose, and mouth) and less salient (out of area of interest) regions, (iii) the presumed occurrence of complementary fixations on the non-informative part of a picture (to test if face processing goes beyond stimulus-driven mechanisms), as well as (iv) the possible face inversion effect on half-faces.

With respect to the left-gaze bias, we expected to find evidence supporting the preference for the left half of the stimuli: more and longer fixations at the left compared to the right informational half, as well as the left compared to the right covered parts.

Our account is based on data (Janik et al., 1978; Itier et al., 2007) suggesting that the eye, nose, and mouth region play a prominent role in face processing (the eyes being the most salient), so we predicted more fixations targeted at these regions compared to other, less relevant areas. In addition, the dominance of the eye (and, less robustly, the nose and the mouth) region is so apparent that we expected to detect this preference in inverted faces as well.

Concerning the complementary fixations, scanning the non-informative (i.e., missing) part of the facial image would mean that visual information processing of faces is not purely stimulus-driven but it involves template-driven recognition mechanisms as well – if these complementary fixations are targeted at the regions corresponding to the most relevant, socially salient regions (eyes, nose, mouth). We believe that finding this kind of fixations would support the template hypothesis.

As to the inversion effect, two competing hypotheses can be put forth: people either spend more time (i.e., fixations) at scanning upright as opposed to inverted faces (they are better at processing normal, upright faces, therefore they fixate them more), or, alternatively, people use up more cognitive effort for perceiving these atypical faces, looking more at inverted compared to upright half-faces (e.g., Barton et al., 2006).

## Study 1

### Materials and Methods

#### Ethics Statement

Research was approved by the Inter-University Psychology Research Ethics Committee (approval number: 2015/23).

#### Participants

Participants were recruited between December 2014 and February 2015. Most of them took part as a course requirement, the remaining were approached at the university (approximately a total of 30 people were contacted). Of the original 24 participants (12 men, 12 women, mean age ± SD = 25.1 ± 3.3 years, age range: 20–30 years), one dropped out as they provided insufficient eye-tracking data. All participants volunteered to take part in the study and provided written consent. Each participant was white, had normal or corrected-to-normal vision and was naive to the experiment’s purpose.

#### Apparatus

Eye movements were monitored using a Tobii T60 XL eye-tracker. The eye tracker performed binocular tracking with the following characteristics: accuracy 0.5°, spatial resolution 0.35°, and eye position sampling frequency of 60 Hz.

The Tobii Studio I-VT (velocity-threshold identification) fixation filter was used to distinguish fixations from saccades. The threshold velocity was set to 30°/s (as recommended by the filter). Eye movement records above the velocity threshold were discarded from the analyses.

#### Stimuli

For stimuli, we used front-view images of the faces of seven white women with neutral expressions, placed on a light background. The images were obtained with permission from the Radboud Faces Database^®^ (RaFD, Langner et al., 2010), and they measured 1019 × 647 pixels.

**FIGURE 1:**
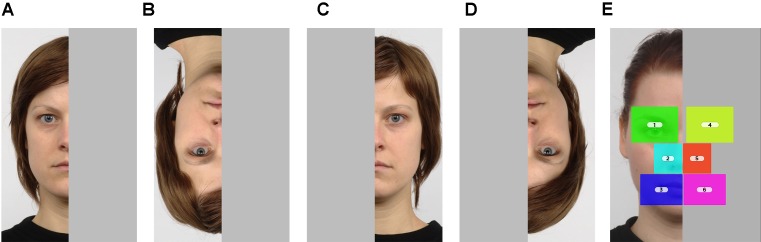
An example of the four stimulus versions presented in the study. **(A)** Upright – left side visible. **(B)** Inverted – left side visible. **(C)** Upright – right side visible. **(D)** Inverted – right side visible. The fixation data were collected for six areas of interest (AOIs) **(E)**: (1) eye visible, (2) nose visible, (3) mouth visible, (4) eye covered, (5) nose covered, (6) mouth covered, as well as the out of AOI areas. The images were obtained from the Radboud Faces Database^®^, with permission (Langner et al., 2010).

The images were edited using Adobe Photoshop 5.1 software. First, for some images, the background regions above the hairline, below the neck line or on the two sides of the face were cropped and the image resized, so that both the length and width of the faces on the seven images became similar in size. Second, the images were vertically cut in half and one half was covered with a gray rectangle. For each image we created two versions, covering either the left or the right side of the image. In the third step, we created an inverted version for each of these images by rotating 180° upside down. This resulted in a total of 28 stimuli, four versions from each of the seven model face: 2 orientations (upright vs. inverted) × 2 visible sides of the image (left vs. right; from now on, by left or right side of the image we mean that from the observer’s point of view) (see Figure 1).

#### Procedure

The stimuli were presented on the 24 inch LCD monitor (resolution: 1920 × 1200 pixels), controlled by a PC located in the same room. Each participant was tested alone in a quiet and darkened room, seated approximately 70 cm from the monitor. The images were presented at 37° of angle. The participants were given no specific, task-related instructions prior to the experiment, except that they needed to watch the images appearing on the computer screen and to look freely wherever they felt like.

The experiment started with a calibration phase using the Tobii’s 5-point calibration procedure. If calibration was successful (i.e., the participant made eye-movements at minimum four calibration points), it was immediately followed by the test phase, otherwise the calibration was repeated until it was successful.

In the test phase, each participant was presented with a unique sequence of eight different stimuli. Every stimulus was presented at the center of the screen for 7 s. Before the half-face stimuli, a short attractor (an animation used for infant studies, depicting a small rotating rattle, with sound) was presented in the center of the screen for 4 s to ensure that the participant’s gaze be directed at the center before the next display appeared.

From the seven model faces, four were chosen for each participant. Each of these four was presented once in upright and once in inverted (upside down) orientation, so all participants were presented with four upright and four inverted stimuli. For both orientations, the left and the right side of the image was visible 2-2 times.

The stimulus sequence was based on the following rules: no more than two stimuli with the same orientation could follow each other; no more than two stimuli with the same side of the image visible could follow each other; two stimuli with the same combination of orientation and visible side of the image (e.g., upright, left side visible) could not follow each other; two stimuli with the same model face could not follow each other; the combination of orientation of the stimulus and visible side of the image in the first trial was counterbalanced across both male and female participants (e.g., four female and four male participants started with an upright stimulus where the left side of the image was visible).

#### Statistical Analyses

##### Data preparation

We defined 6 areas of interest (AOI): 2 stimulus sides (visible, covered) × 3 regions. The three AOIs defined on the visible side were corresponding to the eye, nose and mouth regions. Three AOIs with corresponding size and placement were defined for the covered side of the stimulus (see Figure 1). The sizes of the AOIs were similar but not identical (the nose region being the smallest). All AOIs combined took up less than half of the picture (so the out of AOI area was bigger on both the visible and the covered halves).

In each trial we recorded fixation count (representing the total number of fixations) and fixation duration, separately for each AOI, as well as for out of AOI areas.

##### Fixations targeted at the covered half

To test if template-driven mechanisms are involved in face processing, in the first step we analyzed participants’ tendency to fixate on the covered, non-informative half of the face stimuli. Also, we tested if any characteristics of the participants or of the stimuli affected whether participants fixated on the covered half of the stimulus at all. We used a generalized linear mixed model (GLMM) with a binary score as dependent variable: for each stimulus, participants received score 1 if they fixated at any AOI on the covered half of the stimulus, and score 0 if not. Fixed factors included (1) sex of the participant (male, female); (2) visible side of the image in the first trial (left, right); (3) orientation of the stimulus in the first trial (upright, inverted); (4) model face ID (which model face was presented); (5) visible side of the image (left, right); (6) orientation (upright, inverted). The trial number (1st – 8th) was added as covariate. Participants’ identity was included as random factor to account for the repeated measures structure in the dataset. Non-significant effects were removed from the model in a stepwise manner.

##### Preference for eye, nose and mouth regions

The raw data of fixation count and fixation duration were transformed into relative fixations (percentage of the total fixations) to account for the differences in the total duration and total number of fixations between participants and between stimuli.

To test if participants fixated more often at the more relevant and informative parts of the image (eye, nose, and mouth) compared to the less relevant, out of AOI areas (including the cheek, forehead, hairline, ears, and neck), relative fixations targeted at each of the six AOIs and the out of AOI area were calculated as a percentage of the total fixations targeted at the whole stimulus (i.e., by dividing the fixations at a given region by the total fixations at the whole stimulus, then multiplied by 100).

For the analysis, fixations targeted at the image were grouped into two categories: relevant (summing the six AOIs, corresponding to the eye, nose, and mouth regions of the two image halves) and non-relevant (including all other parts of the image). Relative fixation count and relative fixation duration were included as dependent variables in generalized estimating equations (GEE) models. Fixed factors included (1) visible side of the image (left, right); (2) orientation (upright, inverted), (3) stimulus half (visible, covered); and (4) region (relevant vs. non-relevant). Two-way interactions between region and the other three factors were added to the model to analyze if the preference for face-relevant regions was different for the visible and covered halves of the stimuli, for the upright and inverted stimuli, or when the left and right side of the image was visible. Participants’ identity was included as random factor. Non-significant effects were removed from the model in a stepwise manner. For *post hoc* tests, the Bonferroni method was used.

##### Fixation pattern on the visible and the covered stimulus halves

To test if the pattern of fixations among the facial regions was different for the visible and covered halves of the stimuli, we calculated relative fixations targeted at each of the three AOIs on a given stimulus half as a percentage of the total fixations targeted at that half (i.e., by dividing the fixations at a given AOI by the total fixations targeted at the stimulus half where that AOI was located, then multiplied by 100). If in a given trial, no fixation was targeted on any AOIs on the covered half of the stimuli, we treated them as missing data for the covered half (there was no trial when no fixation was targeted at the AOIs on the visible half of the image).

Relative fixation count and relative fixation duration at each AOI were included as dependent variables in GLMM models. Fixed factors included (1) visible side of the image (left, right); (2) orientation (upright, inverted), (3) stimulus half (visible, covered); and (4) region (eye, nose, mouth).

All two- and three-way interactions were added to the model. Participants’ identity was included as random factor. Non-significant effects were removed from the model in a stepwise manner (backward elimination technique). For *post hoc* tests, the Bonferroni method was used.

All the statistical analyses were conducted with SPSS statistics 22.0 (IBM SPSS Statistics for Windows, Version 22.0. Armonk, NY, United States: IBM Corp.).

### Results

#### Fixations Targeted at the Covered Half

To see if complementary fixations occurred, we analyzed gaze events targeted at the covered half. Three participants never fixated at the covered half of the stimuli in any of the trials. The other 20 participants fixated at the covered half in at least one trial out of the eight (mean: 2.55 trials, SD: 1.53). However, the results showed that neither the characteristics of the participants (sex, visible side of the image in the first trial and orientation of the stimulus in the first trial), nor of the stimuli (which model face was presented; which side of the image was visible; the orientation of the stimulus and the trial number) affected significantly whether or not participants fixated on the covered half (*p* > 0.137 for all at removal).

#### Preference for Eye, Nose and Mouth Regions

First, we tested whether participants fixated more often at the face-relevant parts of the image (eye, nose, and mouth) compared to the non-relevant areas. The relative fixation count and relative fixation duration at each AOI did not differ significantly between the two orientations (upright vs. inverted: count: *p* = 0.115; duration: *p* = 0.278 at removal) and between the two visible sides of the image (right vs. left: count: *p* = 0.405, duration: *p* = 0.153 at removal); neither did we find significant interactions between these factors and the region (count: *p* = 0.126 and *p* = 0.232, duration: *p* = 0.201 and *p* = 0.226 at removal, respectively). We found significant interaction between stimulus half and region (count: Wald χ^2^_1,732_ = 30.889, *p* < 0.001; duration: Wald χ^2^_1,7328_= 34.474, *p* < 0.001). The *post hoc* analyses indicated that participants fixated more and longer at the relevant regions than at the non-relevant regions both on the visible and the covered halves of the image (visible half, count: *p* < 0.001, duration: *p* < 0.001; covered half, count: *p* = 0.002, duration: *p* = 0.017). However, the preference for the relevant regions seems to be higher on the visible half than on the covered half.

In order to find out if there is a hierarchy among the relevant facial regions (i.e., preference for eye, nose and/or mouth areas), we conducted analyses about fixations targeted at the AOIs and their relation to other variables. Similarly to the model described above, the relative fixation count and relative fixation duration at each AOI did not differ significantly between the two orientations (upright vs. inverted), and between the two visible sides of the image (right vs. left) (*p* = 1 at removal for both factors, both variables); neither did we find significant interactions between these factors and the region (count: *p* = 0.315; *p* = 0.300; duration: *p* = 0.217 and 0.413 at removal, respectively). However, again, the interaction between stimulus half and region was significant for both dependent variables (count: Wald χ^2^_2,1098_= 94.643, *p* < 0.001; duration: Wald χ^2^_2,1098_= 90.073, *p* < 0.001).

*Post hoc* tests revealed that on the visible side, participants fixated more often and longer on the eye region than on the nose and mouth regions (*p* < 0.001 for all). However, on the covered half, participants fixated more often and longer on the nose than the eye and mouth regions (*p* < 0.001 for all).

#### Fixation Pattern on the Visible and Covered Stimulus Halves

We found a three-way interaction for the relative fixation duration and relative fixation count: between stimulus half, orientation and region (fixation count: *F*_2,768_= 6.865, *p* = 0.001; fixation duration: *F*_2,768_= 8.959, *p* < 0.001).

To further explore and interpret this three-way interaction, we investigated the two sides of the images separately. These models included orientation, stimulus half and region as main effects, and all two- and three-way interactions.

##### Visible half of the face

**FIGURE 2:**
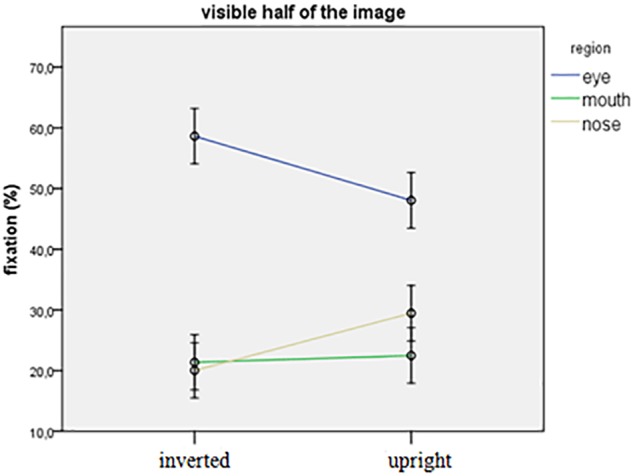
Relative fixation values targeted at the three regions on the visible half of the stimulus.

Figure 2 shows the fixation pattern on the visible half of the picture. We found a significant interaction between orientation and region for both dependent variables (count: *F*_2,543_= 9.354, *p* < 0.001; duration: *F*_2,543_= 9.565, *p* < 0.001). The general pattern of fixation was similar for both orientations: the eye region was fixated more often and longer than the nose and mouth regions (*p* < 0.001 for all). However, the preference for the eye region was stronger in the case of inverted stimuli: participants fixated more often and longer at the eye region but less often and shorter at the nose region of inverted stimuli compared to the upright orientation (eye region, count, duration: *p* = 0.001; nose region: count: *p* = 0.004, duration: *p* = 0.003). No other significant main effects or interactions were found in any of the dependent variables.

##### Covered half of the face

**FIGURE 3:**
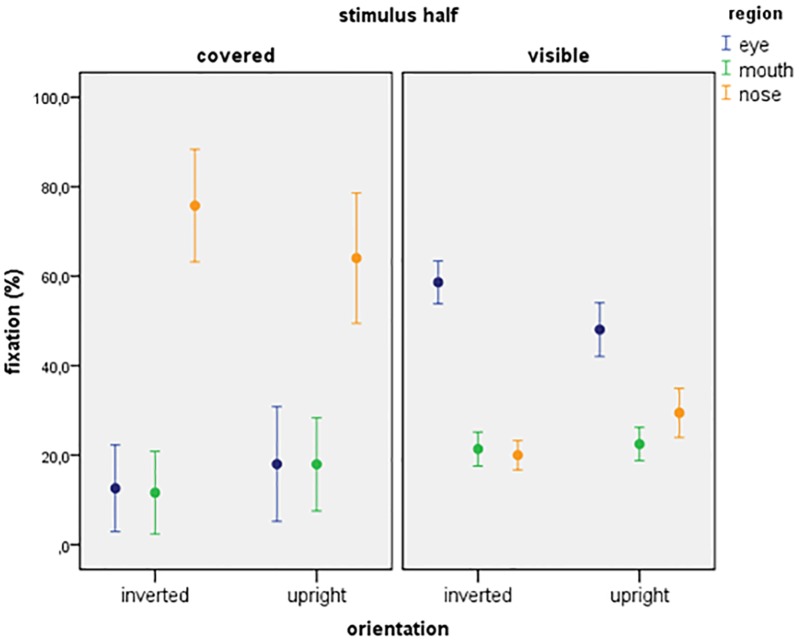
Relative fixation values targeted at the three regions on the covered and the visible side of the stimulus.

Figure 3 depicts the proportion of fixations on the covered half of the stimulus. Here, only the main effect of region was significant (count: *F*_2,228_= 64.063, *p* < 0.001; duration: *F*_2,228_= 56.601, *p* < 0.001). Participants fixated more often and longer at the nose region compared to the eye and the mouth region (*p* < 0.001 for all). No other significant main effects or interactions were found in any of the dependent variables.

##### Fixation heatmaps

**FIGURE 4:**
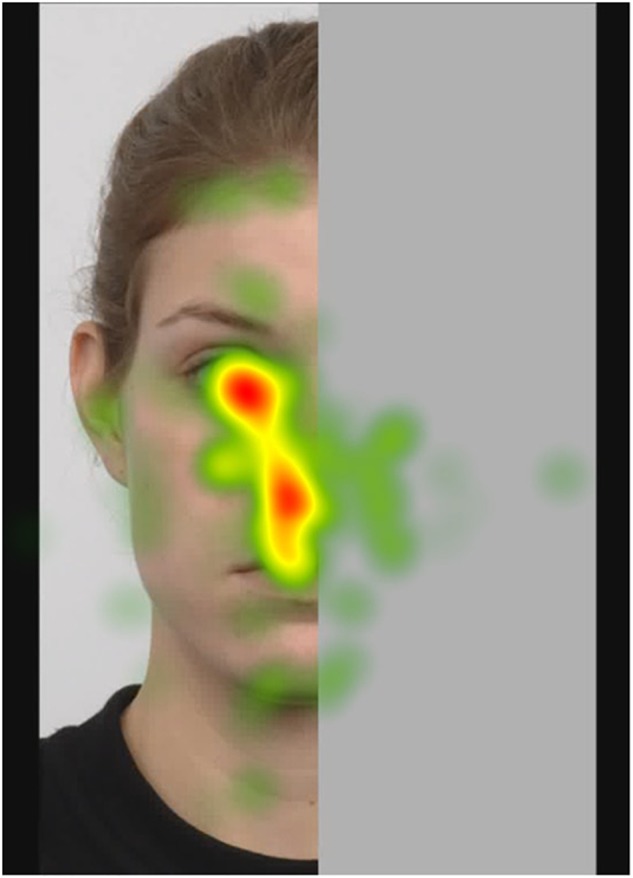
Fixation heatmap of a left upright stimulus.

**FIGURE 5:**
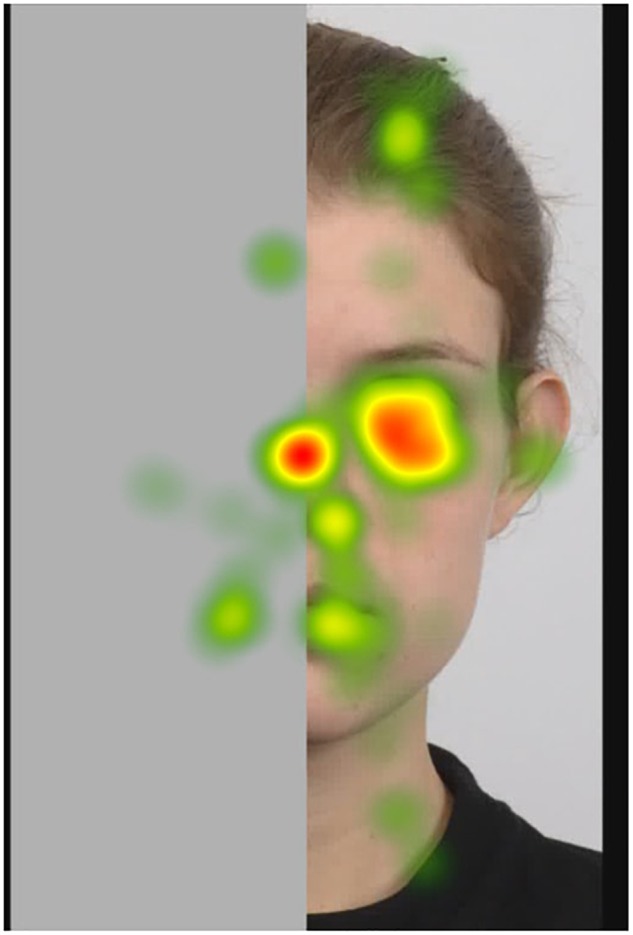
Fixation heatmap of a right upright stimulus.

**FIGURE 6:**
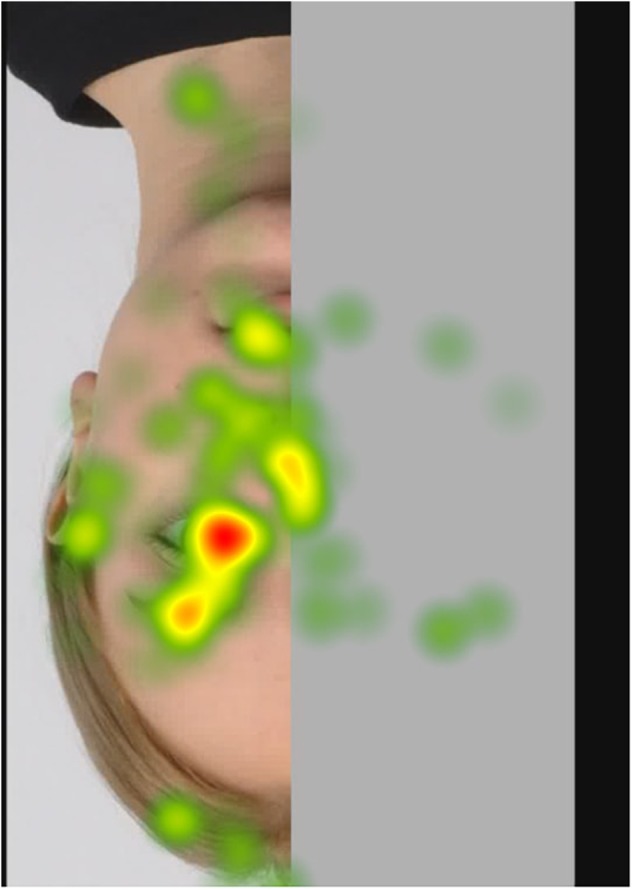
Fixation heatmap of a left inverted stimulus.

**FIGURE 7:**
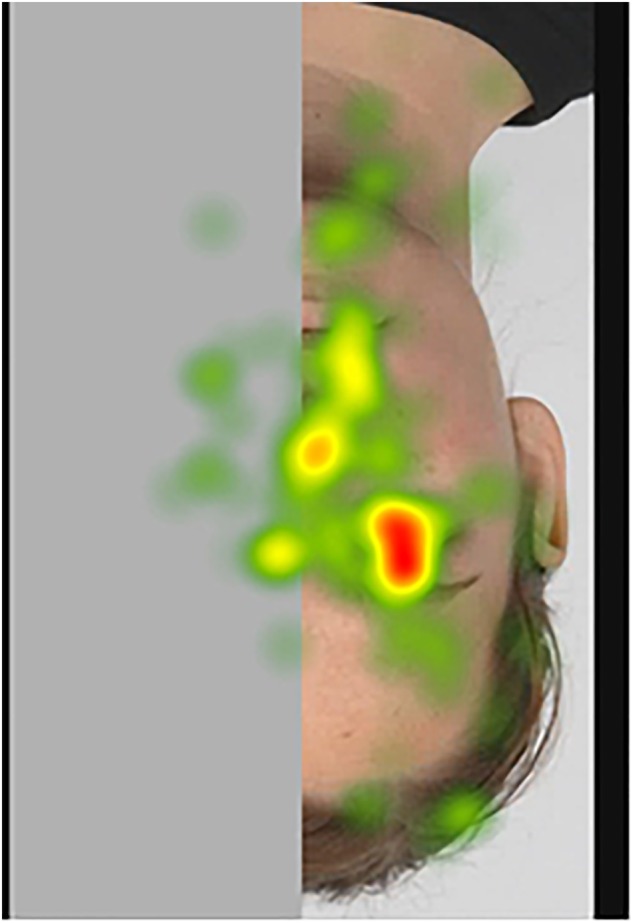
Fixation heatmap of a right inverted stimulus.

Tobii Studio allows for the visualization of the aggregated fixations in the form of fixation heatmaps, which demonstrate the intensity of visual processing (measured by fixation duration) across all participants. Figures 4–7 show these heatmaps for the four types of stimuli.

### Discussion

The purpose of this eye-tracking study was to explore the fixation pattern of adult human participants on both upright and inverted female half-face stimuli in order to gain insight into the typical fixation pattern of scanning half-faces, as well as to test the context-sensitivity (upright vs. inverted position, left vs. right side) and ‘template-drivenness’ (complementary fixations) of face processing in humans.

A majority of participants demonstrated complementary gaze events; 20 out of 23 participants fixated on the non-informative part of the image. They did so in all cases, be the half-face presented on the left or the right side, in upright or inverted orientation. While these fixations on the “virtual” side of the face were limited in number (only 2.5 out of 8 trials on average), they were neither last in the scanning sequence (on average, the first fixation on the covered side occurred at 2.5 s; whereas the less relevant, out of AOI parts of the visible side were fixated at about 4 s), nor randomly placed (see below).

When analyzing different regions of the image, participants fixated at the most informative region (eye, nose, and mouth) of faces longer and more often than at less informative regions, irrespective of the orientation (upright vs. inverted) and the visible side of the face (left vs. right). Our findings are in line with previous studies in which the fixation hierarchy, the dominance of the informative (eye, nose and mouth) regions in visual processing of faces have been robustly demonstrated (e.g., Henderson et al., 2005). These results suggest that the region marked by the eyes, the nose and the mouth is of primary importance, presumably because of the role they play in social situations (e.g., Kaspar and König, 2012) and their physical properties (Farroni et al., 2005). The apparent primacy of this region in both upright and inverted faces demonstrates the robustness of the dominance of the triangular area. Even though the inversion effect may slow down face processing (Haxby et al., 1999), the eyes, the nose and the mouth continue to attract the most fixations. Importantly, similarly to the visible half, the fixations on the covered half were primarily targeted at the corresponding eye, nose and mouth regions. Moreover, in light of the fact that the out of AOI part was larger than the combined AOI regions, these findings point to the direction that complementary fixations are not random but rather follow a pattern which might stem from a template-like face, conveying most information via the region containing the eyes, the nose and the mouth. This internal representation possibly reflects the social significance of faces in general, and the experience we have with upright-oriented facial stimuli. This template can be considered an averaged, abstracted, prototypical face-like stimulus, which serves to influence the processing of visual input, making us predisposed to the perception of this specific class of stimuli. We believe this model is so robust that it may influence fixations even in the absence of certain visual features.

In accordance with our expectations, we found a marked difference between fixations on the visible and the covered halves. Among the three salient areas, the eyes were the ones at which most fixations were targeted on the visible side of the photographs. This finding corroborates the dominance of the eye region (even against the mouth area) demonstrated consistently in the face processing literature (e.g., McKelvie, 1976).

On the covered half, however, participants showed a preference for fixating the nose region. The fact that the nose region attracted more fixations than either the eye or the mouth area on the covered side of the images, is worth exploring. Fixations on the non-informative half were fewer in number and variance as well, compared to the side depicting the half-face. We think that, at least in part, the decreased variance explains the smaller, more circumscribed area of fixations, which, therefore, were mainly focused around the nose region. Being the part that is most central and closest to the facial information, it is conceivable that this is why most fixations on the covered half landed on the corresponding nose area (central bias, e.g., Tatler et al., 2005).

As to the variable *side*, our results were somewhat unexpected. In spite of the fact that the left-gaze bias (a phenomenon that the left side of the face – or visual stimuli in general – typically has advantage over the right side) is consistently demonstrated in the literature (e.g., Siman-Tov et al., 2007; Guo et al., 2009; Coutrot et al., 2016), we did not find a main effect of side (for instance that participants would fixate longer in trials with left half-faces as opposed to trials with right half-faces). This may be because the two halves (informative vs. covered) were perceptually so distinct. But the lack of a clear pattern might also be connected to the fact that the presented side of the face varied as a function of orientation. That is, in upright pictures, the right side of the face was in the left visual field of the observer, whereas in inverted photographs, participants saw the left side of the face in their left visual field. However, we tested if the actual side of the face (i.e., the side from the model’s perspective, not the observer’s) had an effect, but it did not. To see if the left-gaze bias might be present in a more subtle manner, we divided the original eye region into two symmetrical, smaller AOIs, equal in size: the left and the right part. Analyzing the data, an interesting pattern occurred: participants made more fixations at the left side of the eye, if the right side of the face was visible (60.3%), compared to the case when the left side was visible (29.3%) (*p* < 0.001). Regarding the right side of the eye, it was targeted by more fixations if the left side of the face was visible (70.71%), as opposed to when the right side was visible (39.7%) (*p* < 0.001). Also, if the left side of the face was visible, the right half of the eye attracted more fixations (70.71%) than the left side (29.29%) (*p* < 0.001). If the right part of the face was visible, participants made more fixations at the left (60.3%), than at the right half of the eye (39.7%). However, this did not reach statistical significance (*p* = 0.076). These findings can be interpreted from the template narrative: more fixations were directed at the region of the eye that was closer to the covered side (therefore, to the missing features). Thus, it is conceivable that the putative inner representation not only elicited complementary eye movements but it also acted as a magnet, pulling fixations toward the direction of the missing features of the face.

Concerning the inversion phenomenon, there was no significant difference in either fixation count or fixation duration between inverted and upright stimuli. However, inversion did influence looking behavior in a more subtle, interactive way: the nose area on the visible side was scanned more intensively if the face was presented in upright position (compared to inverted), whereas we found a stronger preference for the eye region in inverted photographs (compared to the upright ones). The latter finding is in line with studies highlighting the importance of the eyes in the face inversion effect (Itier et al., 2007). As face inversion disrupts the normal face context, perceiving the eyes (the primary loci of social signals) may need an enhanced level of processing (measured by number and duration of fixations in our study, or by an increase in the amplitude and latency of N170, as in Itier et al., 2007). However, the lack of this effect on the covered side requires further investigation (what may play a role here is that on the covered half, without receiving actual visual information, there are no clear-cut boundaries separating the eye, nose and mouth regions).

In addition to template-related mechanisms, face perception involves feature-based processes as well (Schyns et al., 2002; Farroni et al., 2005). Thus, it is possible that purely physical features (such as saliency) of some image areas correlate or coincide with the most-fixated areas. In order to quantify some physical features of our images, we computed the saliency maps (see e.g., Itti and Koch, 2000) of the four types of the facial stimuli (one picture of each category was analyzed: left upright, right upright, left inverted, right inverted). The saliency computations were done using the Itti-Koch model (MATLAB R2018b software, Image Processing Toolbox and Saliency Toolbox, The MathWorks, Inc., Natick, MA, United States). Then, the saliency maps for each stimulus type were visualized (see Supplementary Figures S1–S4). The visual inspection of these maps indicated that fixations were not accounted for by physical properties of the images, since there was little overlap between the fixation heatmap and the saliency-based maps.

Finally, we ran a receiver operating characteristic (ROC) analysis in order to determine the explanatory power of the Itti-Koch model in terms of the spatial distribution of fixations (i.e., to see how well the saliency map predicts fixations). The area under curve (AUC) scores were 0.07 for the left upright, 0.27 for the right upright, 0.11 for the left inverted and 0.44 for the right inverted picture. Hence, all AUC scores were below 0.50, suggesting that the saliency model was not a good predictor of fixations in our study.

To see if the fixation patterns and the complementary eye movements we found are indeed face-specific, we ran a study using control objects. The motivation was to present stimuli that are symmetrical but whose processing presumably is not based on holistic, template-related mechanisms. Therefore, we chose the following object categories: cars, houses and trees. Even though perception of cars shows some similarity with that of faces (see Windhager et al., 2010), we expected that the visual scanning follows a different pattern for faces and for the other visual objects.

Another rationale for Study 2 was related to the stimulus presentation time frame. We aimed to find out if the complementary fixations occur fast, even when there is only 2.5 s to scan the images.

Thus, differences between Study 1 and Study 2 are the following: different stimulus categories (faces, cars, houses, and trees); shorter stimulus presentation time (2.5 s); and different number of trials per category (for faces, there were three trials per category; for the objects, only one).

## Study 2

### Materials and Methods

#### Ethics Statement

Research was approved by the United Ethical Review Committee for Research in Psychology (EPKEB) (reference number: 2016/075).

#### Participants

Ten adult participants with normal or corrected-to-normal vision took part in this study. They all volunteered to participate, provided written consent, did not receive any compensation, and were naive to the experiment’s purpose. There was no overlap between participants in Study 1 and 2.

#### Apparatus

Same as in Study 1, see detailed description above.

#### Stimuli

Faces were again used from the Radbound Faces Database^®^ (Langner et al., 2010). The other stimuli were schematic, colored images of cars (647 × 480 pixels), houses and trees (647 × 1019), with the same background as the one used for faces. All these stimuli were sequentially presented with the following constraints: no more than two of the same category (face, car, house, tree)/orientation (upright, inverted)/visible side (left, right) could follow each other.

#### Procedure

Same as in Study 1 (see detailed description above), with the following exceptions. Each stimulus was presented for 2.5 s, with a 0.5-s-long fixation cross between stimuli. For faces, there were three stimuli in the same category (e.g., left inverted), whereas for the objects, only one.

#### Statistical Analyses

##### Data preparation

We defined three AOIs. For faces, the “visible eye” was the area surrounding the eyes. For cars and houses, the “eye” was the corresponding feature of the specific category (headlight for cars, window for houses). For trees, an AOI of the same size, in the upper part of the stimuli was defined. For each category, the “covered eye” region was the corresponding area on the covered side (i.e., where the actual feature would be). For the covered half, a bigger AOI called “eye large” was also defined because we anticipated that the fixations may not land on the exact position of the covered eye, only approximate it.

The sizes of the AOIs were as follows:

- cars:
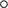
covered eye: 4 × 3 cm
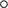
covered eye large: 5.2 × 3.8 cm
- houses:
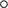
covered eye: 3.8 × 2.6 cm
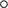
covered eye large: 4.8 × 4 cm
- trees:
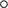
covered eye: 3.8 × 2.6 cm
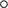
covered eye large: 5.3 × 4 cm

**FIGURE 8:**
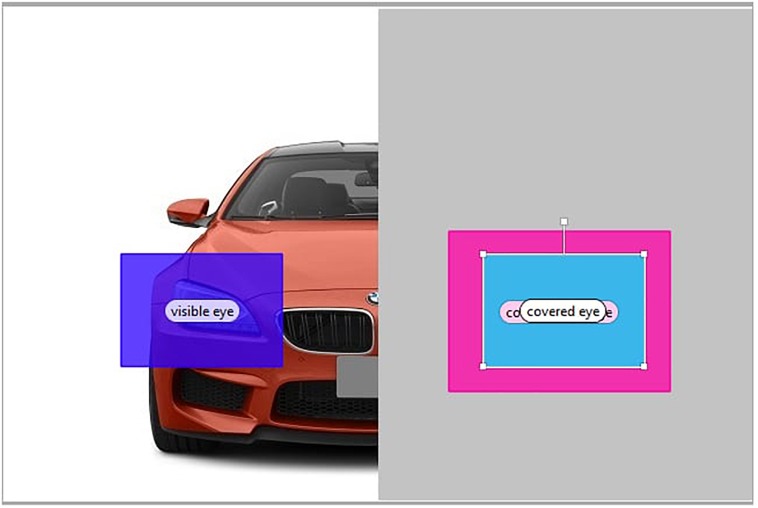
Areas of interest for a car stimulus.

**FIGURE 9:**
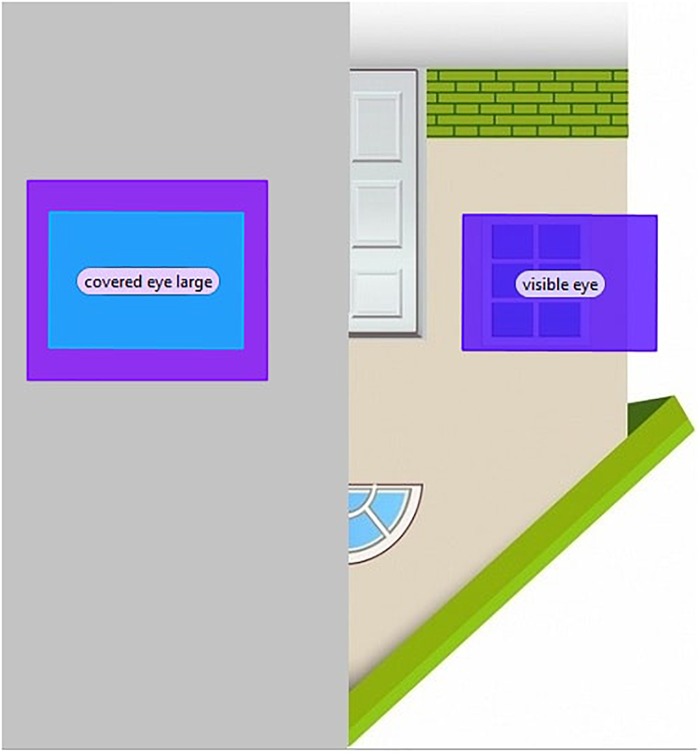
Areas of interest for a house stimulus.

**FIGURE 10:**
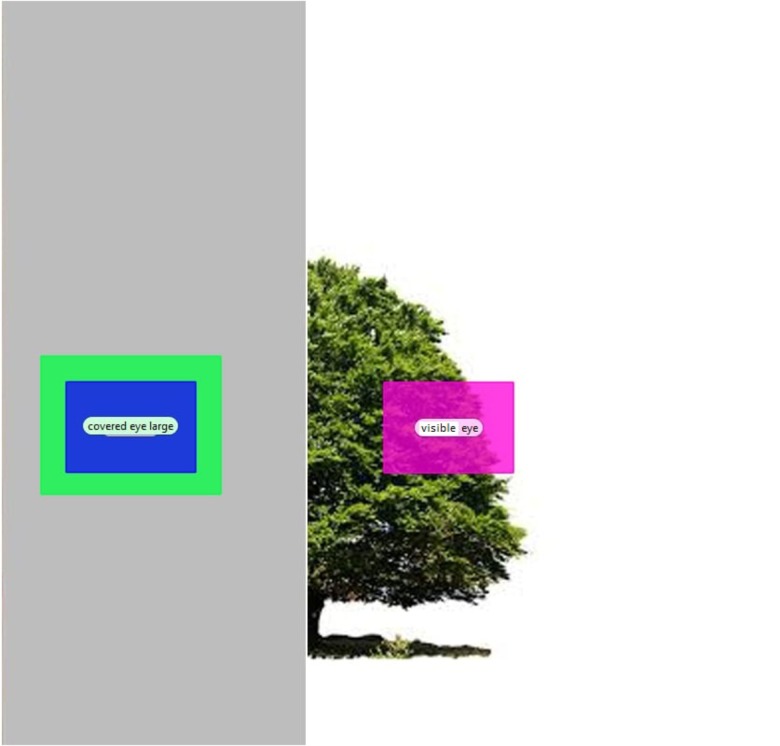
Areas of interest for a tree stimulus.

Figures 8–10 depict the AOIs of the three object categories.

In each trial, we recorded total fixation duration for the AOIs and computed the fixations targeted at the out of AOI as well.

##### Fixations targeted at the covered half

In the first step, we analyzed the participants’ tendency to fixate on the covered, non-informative half of the stimuli. We used a generalized linear mixed model (GLMM) with a binary score as dependent variable: for each stimulus, participants received score 1 if they fixated on the covered half of the stimulus, and score 0 if not. Fixed factors included (1) visible side of the image (left, right), (2) orientation (upright, inverted) and (3) category (faces, cars, houses, trees). Participants’ identity was included as random factor to account for the repeated measures structure in the dataset. Non-significant effects were removed from the model in a stepwise manner.

#### Preference for Eye Region

To test if participants fixated more often at the eyes compared to the less relevant, out of AOI areas, relative fixations targeted at the AOIs and the out of AOI were calculated as a percentage of the total fixations targeted at the whole stimulus (i.e., by dividing the fixations at a given region by the total fixations at the whole stimulus, then multiplied by 100).

Relative fixation duration was included as dependent variable in GEE models. Fixed factors included (1) visible side of the image (left, right); (2) orientation (upright, inverted), (3) stimulus half (visible, covered); (4) region (eye, out of AOI) and (5) category (face, object). Two-way interactions between category and the other factors, and a three-way interaction between stimulus half, region and category were added to the model to analyze if the different features of the stimuli affected the fixation differently for the specific categories. Participants’ identity was included as random factor. Non-significant effects were removed from the model in a stepwise manner. For *post hoc* tests, the Bonferroni method was used.

#### Fixation Pattern on the Visible and the Covered Stimulus Halves

To test if the pattern of fixations among the categories was different for the visible and the covered halves of the stimuli, we calculated relative fixations targeted at the AOIs on a given stimulus half as a percentage of the total fixations targeted at that half (i.e., by dividing the fixations at a given AOI by the total fixations targeted at the stimulus half where that AOI was located, then multiplied by 100).

Relative fixation duration at the AOIs was treated as dependent variable in GEE models. Fixed factors included (1) visible side of the image (left, right); (2) orientation (upright, inverted), (3) stimulus half (visible, covered); (4) region (eye, out of AOI) and (5) category (face, object).

Two-way interactions between category and the other factors, and a three-way interaction between stimulus half, region and category were added to the model. Participants’ identity was included as random factor. Non-significant effects were removed from the model in a stepwise manner (backward elimination). For *post hoc* tests, the Bonferroni method was used.

To see if the specific object categories elicit different fixation patterns, we ran similar analyses on the object categories.

All statistical analyses were conducted with SPSS statistics 22.0 (IBM SPSS Statistics for Windows, Version 22.0. Armonk, NY, United States: IBM Corp.).

### Results

#### Fixations Targeted at the Covered Side

We found a main effect of side: if the image was presented on the left side, participants made eye movements at the covered side in more cases, as opposed to if it was presented on the right side (*F*_1,238_= 8.8, *p* = 0.003). Also, regarding stimulus category, the results show that participants tended to look at the covered side in more cases for faces, compared to the objects. However, this did not reach statistical significance (*p* = 0.088).

#### Preference for the Eye Region

There was a significant interaction between stimulus half and region (small eye: Wald χ^2^= 8.9, *p* = 0.003; large eye: Wald χ^2^= 14.4, *p* < 0.001): the out of AOI was fixated more on both the visible and the covered halves compared to the eye, and both visible regions were fixated more than their covered counterparts (*p* < 0.001 for all).

We also found a significant interaction between stimulus half and category (Wald χ^2^= 7.7, *p* = 0.006): in both categories, the visible side was fixated more than the covered half (*p* < 0.001 for both). However, the visible side attracted more fixations for faces (compared to objects), and the covered side received more fixations for objects (compared to faces) (*p* = 0.006 for both).

Another significant interaction was found between region and object category (small eye: Waldχ^2^= 6.2, *p* = 0.013; large eye: Wald χ^2^= 10.6, *p* = 0.001): while in both categories, the out of AOI was fixated more than the eye (*p* = 0.004; *p* < 0.001 for faces and objects, respectively), the eye region was fixated more in the case of faces (compared to objects), and the out of AOI was fixated more for objects (compared to faces) (*p* = 0.013).

#### Fixation Pattern

For the small eye region, there was a significant interaction between stimulus half and region (Waldχ^2^= 8.1, *p* = 0.005). While in both stimulus halves, the out of AOI was fixated more than the eye (*p* < 0.001 for both), the eye region was fixated more on the visible side (compared to the covered half), whereas the out of AOI attracted more fixations on the covered side (*p* = 0.005 for both).

For the small eye region, we found a significant interaction between region and category (Waldχ^2^= 5, *p* = 0.026). While in both categories, the out of AOI was fixated more than the eye (*p* < 0.001 for both), the eye region was fixated more in the case of faces (compared to objects), and the out of AOI was fixated more for objects (compared to faces) (*p* = 0.026).

For the large eye region, we found a significant, three-way interaction between stimulus half, region and category (Waldχ^2^= 36.5, *p* < 0.001). On both the visible and the covered sides, the eye was fixated more for faces than for objects (*p* = 0.021, *p* < 0.001 for the visible and the covered halves, respectively). The only case where the eyes received more relative fixations than the out of AOI was the covered eye region of faces (*p* < 0.001). For faces, the eye attracted more fixations on the covered side (compared to the visible side, *p* < 0.001); for objects, the eye on the visible side tended to receive more fixations than the eye on the covered half (*p* = 0.077).

#### Fixations on the Different Object Categories

##### Fixations on the covered side

There was no significant difference between the three object categories in whether the participants fixated on the covered side of the stimuli or not (*F*_2,116_= 0.74, *p* = 0.48).

##### Preference for the eye region

We found a significant three-way interaction between stimulus half, region and object category (Wald χ^2^= 20.2, *p* < 0.001). The visible eye region was fixated less, the out of AOI more for trees, compared to the other categories (cars: *p* = 0.004, *p* < 0.001; houses: *p* < 0.001 for both). However, no such difference was found regarding the covered side (*p* > 0.313). Moreover, in cars and houses, both the eye and the out of AOI region were fixated more on the visible side than on the covered side (*p* < 0.001 for all), but in the case of trees, no difference was found between the eye region on the visible and the covered sides (*p* = 0.146).

##### Fixation pattern

Another significant three-way interaction was found between stimulus half, region and object category (Waldχ^2^= 25.7, *p* < 0.001). The visible eye region was fixated less, the out of AOI more for trees, compared to the other categories (cars: *p* = 0.003, *p* < 0.001; houses: *p* = 0.003, *p* < 0.001). However, no such difference was found regarding the covered side (*p* > 0.313). Moreover, in cars and houses, the eye region was fixated more on the visible side and the out of AOI on the covered half (*p* = 0.008 for all), whereas in the case of trees, no difference was found between the visible and the covered sides either in the eye region or in the out of AOI (*p* = 0.945).

##### Heatmaps

**FIGURE 11:**
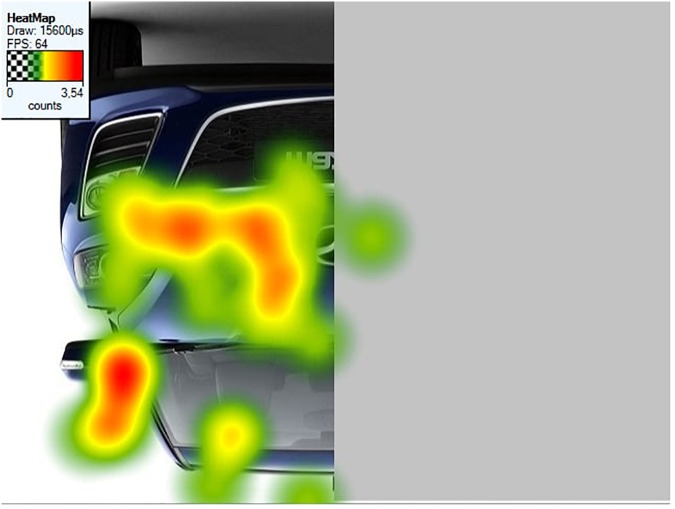
Fixation heatmap of a left inverted car stimulus.

**FIGURE 12:**
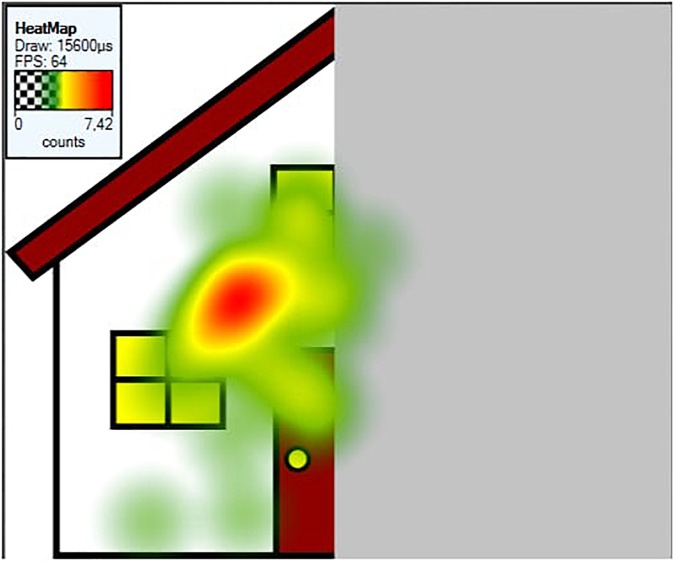
Fixation heatmap of a left upright house stimulus.

**FIGURE 13:**
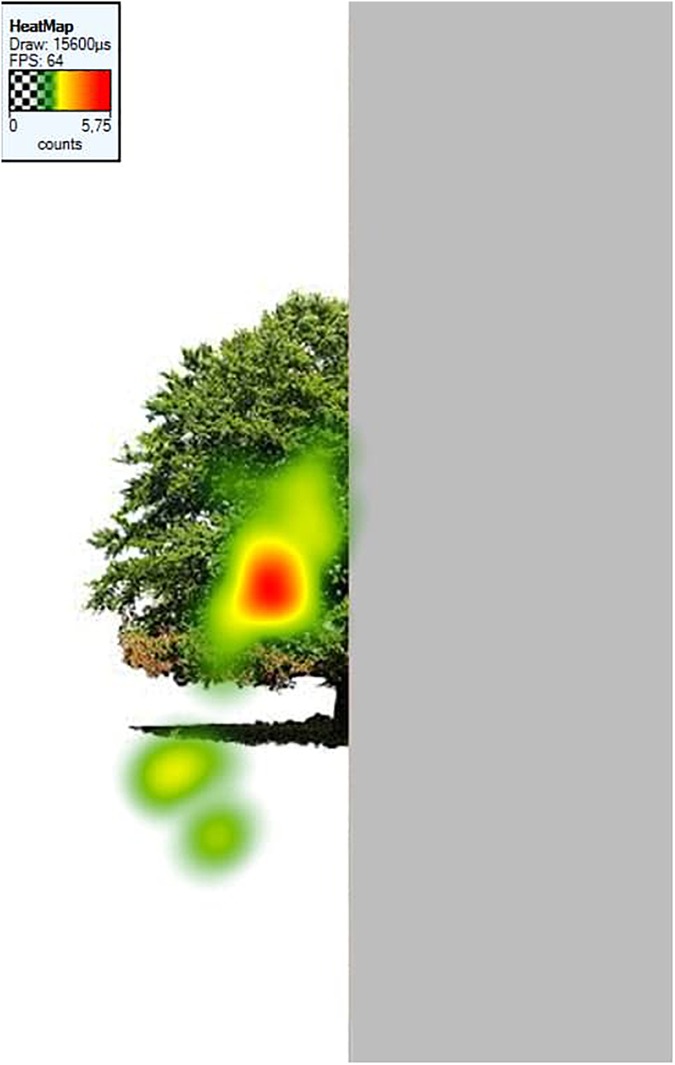
Fixation heatmap of a left upright tree stimulus.

Figures 11–13 show the fixation heatmaps (fixation durations of all participants) of three examples of the object categories.

### Discussion

Taken together, the findings of Study 2 are in line with the face-specific, template-based, eye region-focused fixation pattern hypothesis. First, whether participants made fixations at all at the covered side, was affected by stimulus category: they tended to look at the covered part in more cases if the presented stimuli were faces. Second, there was indeed a preference for the eye region: it received more fixations in faces, as opposed to the objects. Regarding the objects, the out of AOI attracted more fixations, than in faces: fixations were less circumscribed, more broadly distributed. Third, and crucial to our approach, we found that the eye region was fixated more in faces on the covered side as well. Importantly, the only case where the eyes received more relative fixations than the out of AOI was the covered eye region of faces (*p* < 0.001).

Concerning the different object categories, the arbitrarily defined *eye* region of the trees attracted fewer fixations than the other categories. This may have to do with the fact that both cars and houses have eye-like features (headlights and windows, respectively) that serve as a preferred destination for eye movements. In addition, for houses and cars, the *eye* region was fixated more intensively on the visible side, whereas on the covered side, it was the out of AOI that attracted more fixations. This points to the direction that even if there are eye movements on the covered side of the object stimuli, they are not systematic, they do not follow a virtual, complete stimuli, based on the expected arrangement of the features.

### Future Directions and Conclusion

A possible direction for future studies may involve a shift away from the free viewing paradigm, as the task itself may affect the characteristics of scanning (about task-driven effects, see Malcolm et al., 2008). Since our aim was to explore the spontaneous, “unconstrained” looking behavior, in our instruction, we encouraged participants to look freely wherever they felt like. However, it is possible that, in a recognition paradigm, for instance, we may have somewhat different results.

To conclude, we believe that our findings add to the vast body of face perception literature concerning the primal role of the region marked by the eyes, the nose and the mouth. The complementary fixations we found on the covered halves of the pictures indicate that face processing involves template-related mechanisms as well. Study 2 confirmed that these eye movements occur more systematically for faces as opposed to objects. Future research should attempt to further refine and analyze this effect.

## Author Contributions

BT, JT, KO, FE, and ÁG contributed to concept and design. ÁG did the data acquisition. BT, AG, IK, and ÁG analyzed and interpreted the data. ÁG and BT drafted the article. All authors revised the work.

## Conflict of Interest Statement

The authors declare that the research was conducted in the absence of any commercial or financial relationships that could be construed as a potential conflict of interest.

## Footnotes

1 We refer to the terms “analytical,” “feature-based” and ”part-based” as processes that do no involve the integrative component of holistic processes.

2 From now on, we will be using the terms “non-informative,” “empty” and “covered” synonymously, as they all refer to the same part of the stimuli, i.e., the half which does not represent facial information.

## Supplementary Material

FIGURE S1 Saliency heatmap of a left upright stimulus.

FIGURE S2 Saliency heatmap of a right upright stimulus.

FIGURE S3 Saliency heatmap of a left inverted stimulus.

FIGURE S4 Saliency heatmap of a right inverted stimulus.
