# Supplementary figures and images for: Visual Fixation Patterns During Viewing of Half-Face Stimuli in Adults: An Eye-Tracking Study
Source: Front Psychol. 2018 Dec 11;9:2478. doi: 10.3389/fpsyg.2018.02478 (PMC6297881; doi:10.3389/fpsyg.2018.02478)

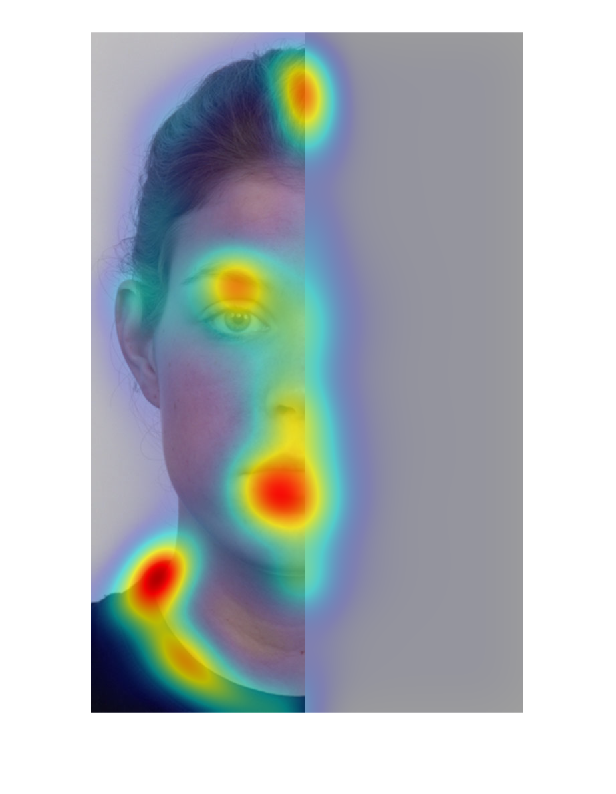

Supplement: FIGURE S1 — Saliency heatmap of a left upright stimulus. [file Image_1.tif]

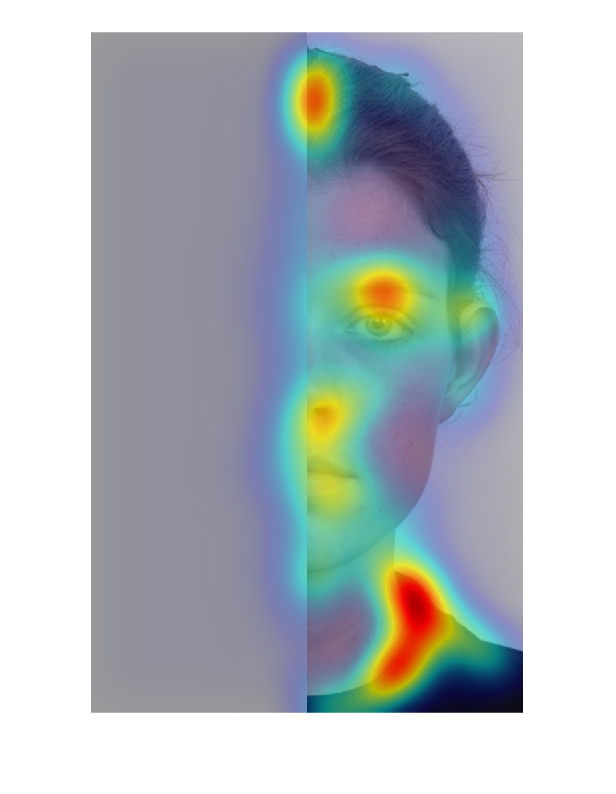

Supplement: FIGURE S2 — Saliency heatmap of a right upright stimulus. [file Image_2.tif]

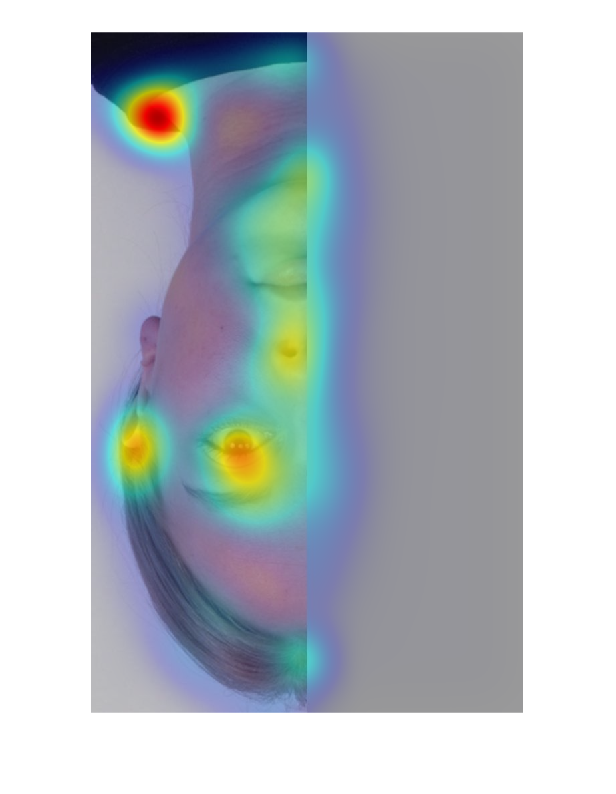

Supplement: FIGURE S3 — Saliency heatmap of a left inverted stimulus. [file Image_3.tif]

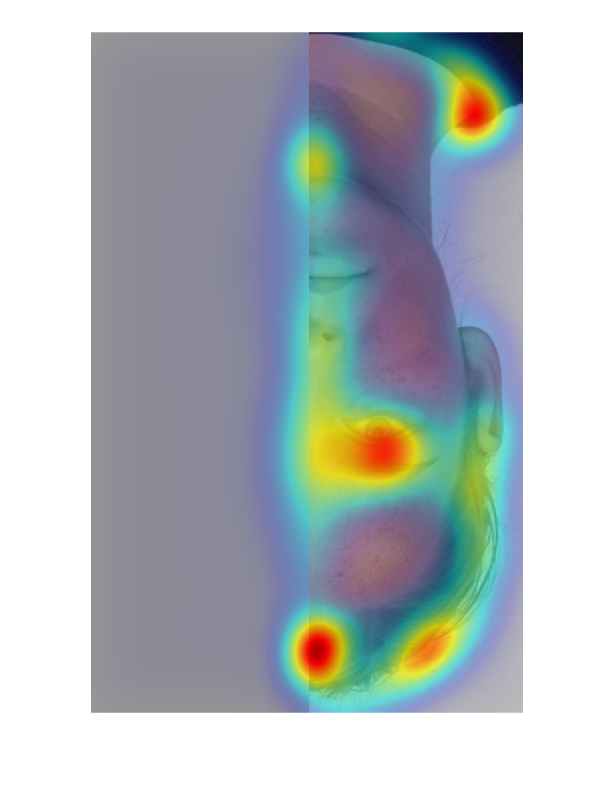

Supplement: FIGURE S4 — Saliency heatmap of a right inverted stimulus. [file Image_4.tif]
